# Supplementary material for: Fasting‐induced liver GADD45β restrains hepatic fatty acid uptake and improves metabolic health
Source: EMBO Mol Med. 2016 May 3;8(6):654–69. doi: 10.15252/emmm.201505801 (PMC4888855; doi:10.15252/emmm.201505801)
Supplement: Supplementary file 1 — Appendix [file EMMM-8-654-s001.pdf]

Table of contents

Appendix Figure S1

Appendix Figure S2

Appendix Figure S3

Appendix Table 1

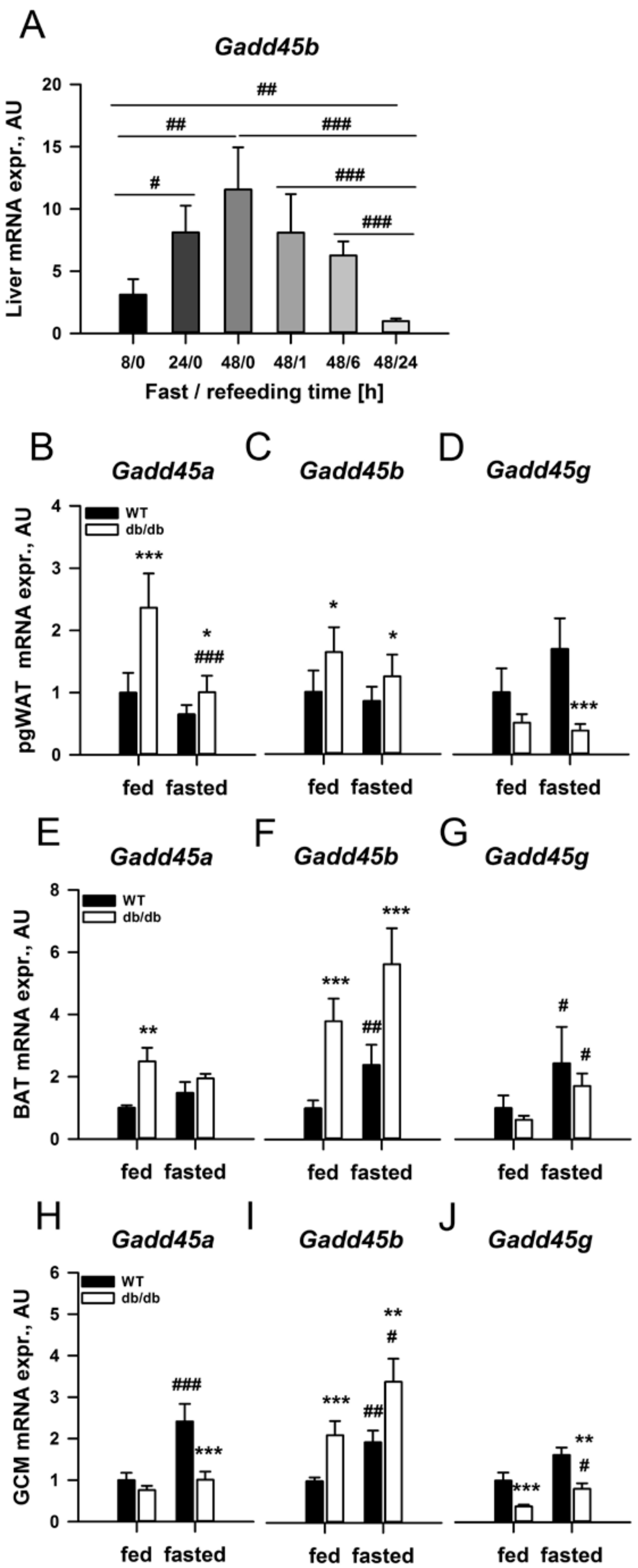

A
Gadd45b
Liver mRNA expr., AU
0
5
10
15
20
#
##
##
###
###
###
8/0
24/0
48/0
48/1
48/6
48/24
Fast / refeeding time [h]
B
Gadd45a
C
Gadd45b
D
Gadd45g
pgWAT mRNA expr., AU
WT
db/db
***
*
###
*
*
***
fed
fasted
E
Gadd45a
F
Gadd45b
G
Gadd45g
BAT mRNA expr., AU
**
***
##
***
#
#
fed
fasted
H
Gadd45a
I
Gadd45b
J
Gadd45g
GCM mRNA expr., AU
###
***
***
##
**
#
***
**
#
fed
fasted

Figure S1. Liver hepatocyte GADD45b expression is consistently dysregulated upon starvation in several mouse models of metabolic dysfunction. Male, 12wk old C57Bl/6J mice were fasted for 8, 24, or 48h or fasted for 48h and subsequently refed for 1, 6, or 24h. Liver *Gadd45b* mRNA expression was measured (A) (n = 4/group). Male, 12wk wildtype (WT; C57bl/6J) or obese/diabetic (*db/db*; BKS.Cg-m+/+ Lepr DB/J) were fed *ad libitum* (fed) or fasted for 24h (fasted) (n=4/group). mRNA expression of Growth Arrest and DNA Damage inducible 45 alpha (*Gadd45a*; B, E, H), beta (*Gadd45b*; C, F I) and gamma (*Gadd45g*; D, G, J) was measured from perigonadal white adipose (B-D), intrascapular brown adipose (E-G) and gastrocnemius complex muscle (H-J). Data are mean ± SEM. Effect of genotype/age/fraction, * $p < 0.05$, ** $p < 0.01$, *** $p < 0.001$. Effect of nutritional state: # $p < 0.05$, ## $p < 0.01$, ### $p < 0.001$. The statistical test used and respective p-value outputs can be found in Appendix Table S1.

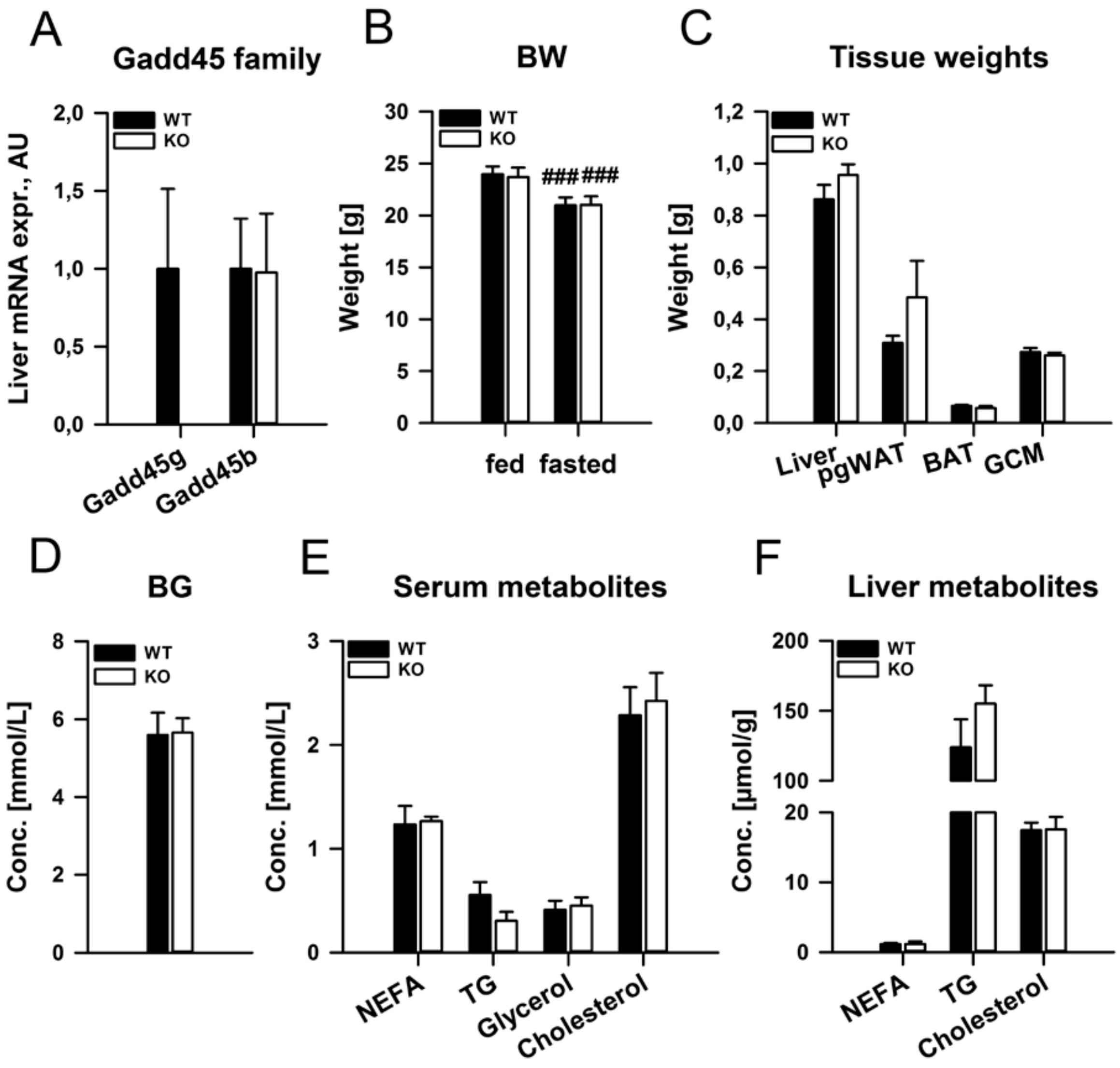
A
Gadd45 family
Liver mRNA expr., AU
2,0
1,5
1,0
0,5
0,0
WT
KO
Gadd45g
Gadd45b
B
BW
Weight [g]
30
25
20
15
10
5
0
###
###
fed
fasted
C
Tissue weights
Weight [g]
1,2
1,0
0,8
0,6
0,4
0,2
0,0
Liver
pgWAT
BAT
GCM
D
BG
Conc. [mmol/L]
8
6
4
2
0
E
Serum metabolites
Conc. [mmol/L]
3
2
1
0
NEFA
TG
Glycerol
Cholesterol
F
Liver metabolites
Conc. [µmol/g]
200
150
100
20
10
0
NEFA
TG
Cholesterol

Figure S2. Systemic GADD45γ deletion does not affect metabolic homeostasis during fasting. Female, GADD45γ+/+ (WT) or GADD45γ-/- (KO) were fasted for 24h (fasted) (n = 5/group) with blood samples taken before and during fasting. Liver mRNA expression of *Gadd45* family members (A). Body weight (B) before and after fasting and fasted tissue weights (C) including perigonadal white adipose, brown adipose and gastrocnemius complex muscle tissues. Blood glucose (D) as well as serum (E) and liver (F) metabolite concentrations, including non-esterified fatty acids, triglycerides, glycerol and cholesterol. Data are mean ± SEM. Effect of genotype, * $p < 0.05$, ** $p < 0.01$, *** $p < 0.001$. Effect of nutritional state: # $p < 0.05$, ## $p < 0.01$, ### $p < 0.001$. The statistical test used and respective p-value outputs can be found in Appendix Table S1.

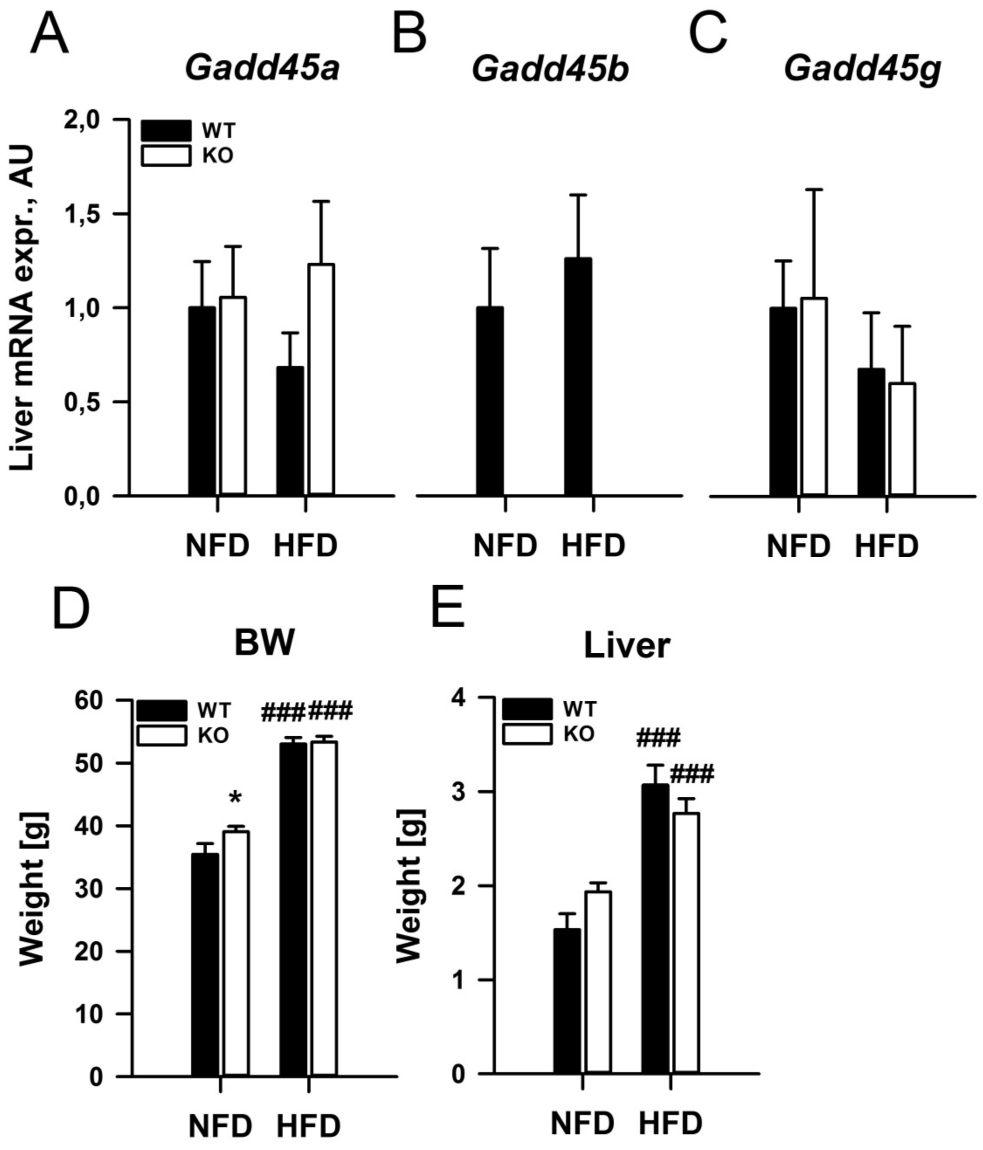

A
Gadd45a
B
Gadd45b
C
Gadd45g
Liver mRNA expr., AU
2,0
1,5
1,0
0,5
0,0
WT
KO
NFD
HFD
D
BW
Weight [g]
60
50
40
30
20
10
0
*
######
E
Liver
4
3
2
1
###

Figure S3. Effects of chronic dietary treatment on metabolic homeostasis in mice with systemic GADD45β deletion. GADD45β +/+ (WT) or GADD45β -/- (KO) were chronically fed a normal- (NFD) or high (HFD) fat diet (n= 6-9/group) and sacrificed in the *ad libitum* fed state. Liver mRNA expression of *Gadd45* family members (A-C). Body weight (D), and liver mass (F). Data are mean ± SEM. Effect of genotype, * $p < 0.05$, ** $p < 0.01$, *** $p < 0.001$. Effect of nutritional state: # $p < 0.05$, ## $p < 0.01$, ### $p < 0.001$. The statistical test used and respective p-value outputs can be found in Appendix Table S1.

Appendix Table S1. P values from statistical tests. NP: not performed.

	Main effects		Interactions	Post-hoc testing				test performed
**Figure 1**	**nutrition**	**Genotype/strain/age**		**fed**	**fasted**	**wt/NZB/young**	**db/NZO/aged**	
				**wt/NZB/ young vs db/NZO/aged**	**wt/NZB/ young vs db/NZO/aged**	**fed vs fasted**	**wt/NZB/ young vs db/NZO/aged**	
**A**	0.004	0.043	0.033	0.233	0.033	0.014	0.031	Two-way ANOVA, Holm-Sidak post-hoc test
**B**	0.158	0.243	0.012	0.042	0.016	0.038	0.025	Two-way ANOVA, Holm-Sidak post-hoc test
**C**	>0.001	0.078	0.046	0.672	0.043	>0.001	>0.001	Two-way ANOVA, Holm-Sidak post-hoc test
**D**	0.077	0.011	0.003	0.088	>0.001	>0.001	0.342	Two-way ANOVA, Holm-Sidak post-hoc test
**E**	0.006	0.044	>0.001	0.279	>0.001	>0.001	0.149	Two-way ANOVA, Holm-Sidak post-hoc test
**F**	0.047	0.027	0.044	0.389	0.033	0.023	0.231	Two-way ANOVA, Holm-Sidak post-hoc test
**G**	0.090	0.113	0.009	0.044	0.028	0.031	0.428	Two-way ANOVA, Holm-Sidak post-hoc test
**H**	>0.001	0.222	0.031	0.385	0.024	>0.001	0.008	Two-way ANOVA, Holm-Sidak post-hoc test
**I**	0.088	0.197	0.027	0.179	0.008	0.009	0.527	Two-way ANOVA, Holm-Sidak post-hoc test
**J**	0.012	0.333	0.021	0.428	>0.001	>0.001	0.200	Two-way ANOVA, Holm-Sidak post-hoc test
**K**	0.009	0.198	0.012	0.371	>0.001	>0.001	>0.001	Two-way ANOVA, Holm-Sidak post-hoc test
**L**	0.021	0.033	0.028	>0.001	>0.001	0.008	0.006	Two-way ANOVA, Holm-Sidak post-hoc test
**M**	>0.001	0.081	0.009	0.287	>0.001	>0.001	>0.001	Two-way ANOVA, Holm-Sidak post-hoc test
**N**	0.031	0.019	0.003	0.471	>0.001	0.006	0.197	Two-way ANOVA, Holm-Sidak post-hoc test
**O**	0.002	0.208	0.019	0.309	>0.001	>0.001	0.06	Two-way ANOVA, Holm-Sidak post-hoc test
**P**	0.005	0.081	0.044	0.618	0.007	0.021	0.009	Two-way ANOVA, Holm-Sidak post-hoc test
**Q**	0.002	0.194	0.049	0.777	0.418	0.033	0.028	Two-way ANOVA, Holm-Sidak post-hoc test
**R**	0.018	0.090	0.037	0.447	>0.001	0.088	0.006	Two-way ANOVA, Holm-Sidak post-hoc test
	**NEFA**	**Genotype**		**BSA**	**BSA-NEFA**	**wt**	**db/db**	
				**wt vs db/db**	**wt vs db/db**	**BSA vs NEFA**	**BSA vs NEFA**	
**S**	0.033	0.011	0.032	0.009	>0.001	0.038	0.008	Two-way ANOVA, Holm-Sidak post-hoc test

**Figure EV1**	**nutrition**	**Genotype/strain/age**		**fed**	**fasted**	**wt/NZB/young**	**db/NZO/aged**	
				**wt/NZB/ young vs db/NZO/aged**	**wt/NZB/ young vs db/NZO/aged**	**fed vs fasted**	**fed vs fasted**	
**A**	0.001	0.005	0.008	0.011	0.023	0.021	0.018	Two-way ANOVA, Holm-Sidak post-hoc test
**B**	0.009	0.222	0.014	0.398	0.121	>0.001	>0.001	Two-way ANOVA, Holm-Sidak post-hoc test
**C**	0.030	0.281	0.039	0.444	0.319	0.007	0.018	Two-way ANOVA, Holm-Sidak post-hoc test
**D**	0.039	0.315	0.049	0.199	0.551	0.051	0.009	Two-way ANOVA, Holm-Sidak post-hoc test
**E**	0.009	0.008	0.007	0.040	0.044	0.033	0.028	Two-way ANOVA, Holm-Sidak post-hoc test
**F**	0.033	0.071	0.088	NP	NP	NP	NP	Two-way ANOVA, Holm-Sidak post-hoc test
**G**	0.055	0.071	0.033	0.388	0.021	0.081	0.050	Two-way ANOVA, Holm-Sidak post-hoc test
**H**	0.221	0.038	0.111	NP	NP	NP	NP	Two-way ANOVA, Holm-Sidak post-hoc test
**I**	0.002	0.091	0.021	0.424	>0.001	>0.001	0.033	Two-way ANOVA, Holm-Sidak post-hoc test
**J**	0.379	0.559	0.394	NP	NP	NP	NP	Two-way ANOVA, Holm-Sidak post-hoc test
**K**	0.021	0.189	0.172	NP	NP	NP	NP	Two-way ANOVA, Holm-Sidak post-hoc test
**L**	0.465	0.444	0.451	NP	NP	NP	NP	Two-way ANOVA, Holm-Sidak post-hoc test

**Figure 2**	**nutrition**	**Genotype/strain/age**		**fed**	**fasted**	**wt/NZB/young**	**db/NZO/aged**	
				**wt/NZB/ young vs db/NZO/aged**	**wt/NZB/ young vs db/NZO/aged**	**fed vs fasted**	**fed vs fasted**	
**A**	0.033	0.066	0.031	0.111	>0.001	0.008	0.189	Two-way ANOVA, Holm-Sidak post-hoc test
**B**	0.003	0.004	0.008	0.089	>0.001	>0.001	0.009	Two-way ANOVA, Holm-Sidak post-hoc test
**C**	0.069	0.077	0.002	0.491	>0.001	>0.001	0.321	Two-way ANOVA, Holm-Sidak post-hoc test
**D**	0.444	0.111	0.044	0.099	0.048	0.122	0.366	Two-way ANOVA, Holm-Sidak post-hoc test
**E**	0.033	0.008	0.021	0.088	0.033	0.007	0.086	Two-way ANOVA, Holm-Sidak post-hoc test
**F**	0.005	0.199	0.033	0.077	0.336	0.021	0.009	Two-way ANOVA, Holm-Sidak post-hoc test
**G**	>0.001	0.111	0.028	0.217	>0.001	>0.001	>0.001	Two-way ANOVA, Holm-Sidak post-hoc test
**H**	0.033	0.041	0.015	0.039	>0.001	>0.001	0.199	Two-way ANOVA, Holm-Sidak post-hoc test
**I**	0.044	0.056	0.027	0.035	0,009	>0.001	0.289	Two-way ANOVA, Holm-Sidak post-hoc test
**J**	0.098	0.181	>0.001	0.389	>0.001	>0.001	0.225	Two-way ANOVA, Holm-Sidak post-hoc test
**K**	0.153	0.210	>0.001	0.255	0.008	0.005	0.351	Two-way ANOVA, Holm-Sidak post-hoc test
**L**	0.073	0.222	>0.001	0.400	0.007	0.006	0.492	Two-way ANOVA, Holm-Sidak post-hoc test

**Figure S1**								
	**8/0 vs 24/0**	**8/0 vs 48/0**	**8/0 vs 48/24**	**48/24 vs 48/0**	**48/24 vs 48/1**	**48/24 vs 48/6**		
**A**	0.039	0.007	0.002	>0.001	>0.001	>0.001		One-way ANOVA, Holm-Sidak post-hoc test
	**nutrition**	**Genotype/strain/age**		**fed**	**fasted**	**wt/NZB/young**	**db/NZO/aged**	
				**wt/NZB/ young vs db/NZO/aged**	**wt/NZB/ young vs db/NZO/aged**	**fed vs fasted**	**fed vs fasted**	
**B**	0.033	0.008	0.021	>0.001	0.038	0.222	>0.001	Two-way ANOVA, Holm-Sidak post-hoc test
**C**	0.111	0.012	0.049	0.038	0.044	0.219	0.200	Two-way ANOVA, Holm-Sidak post-hoc test
**D**	0.288	0.231	0.046	0.051	>0.001	0.191	0.099	Two-way ANOVA, Holm-Sidak post-hoc test
**E**	0.121	0.037	0.039	0.009	0.318	0.298	0.194	Two-way ANOVA, Holm-Sidak post-hoc test
**F**	0.030	>0.001	0.022	>0.001	>0.001	0.008	0.099	Two-way ANOVA, Holm-Sidak post-hoc test
**G**	0.007	0.055	0.044	0.111	0.192	0.035	0.032	Two-way ANOVA, Holm-Sidak post-hoc test
**H**	0.288	0.391	0.014	0.399	>0.001	>0.001	0.404	Two-way ANOVA, Holm-Sidak post-hoc test
**I**	0.010	0.009	0.009	>0.001	0.008	0.007	0.020	Two-way ANOVA, Holm-Sidak post-hoc test
**J**	0.066	0.004	0.017	>0.001	0.007	0.097	0.046	Two-way ANOVA, Holm-Sidak post-hoc test

Figure3								
	nutrition	genotype		pre-fast vs fast	pre-fast vs. refed	fast vs refed		
A	>0.001	0.398	0.021	>0.001	0.044	>0.001		Two-way RM ANOVA, Holm-Sidak post-hoc test
B	>0.001	0.289	0.012	>0.001	0.181	>0.001		Two-way RM ANOVA, Holm-Sidak post-hoc test
C	>0.001	0.455	0.007	>0.001	0.233	>0.001		Two-way RM ANOVA, Holm-Sidak post-hoc test
	nutrition	genotype		fed	fasted	genotype	genotype	
				genotype	genotype	fed vs fasted	fed vs fasted	
D	>0.001	0.033	0.044	0.321	0.008	>0.001	>0.001	Two-way ANOVA, Holm-Sidak post-hoc test
E	>0.001	0.358	0.007	0.243	0.198	>0.001	>0.001	Two-way ANOVA, Holm-Sidak post-hoc test
F	0.055	0.256	0.169	NP	NP	NP	NP	Two-way ANOVA, Holm-Sidak post-hoc test
G		0.009						
	time	genotype		0	90	180	270	
				genotype	genotype	genotype	genotype	
H	>0.001	0.007	>0.001	0.288	>0.001	0.098	0.213	Two-way RM ANOVA, Holm-Sidak post-hoc test
I	0.009	0.032	>0.001	0.555	0.008	>0.001	0.121	Two-way RM ANOVA, Holm-Sidak post-hoc test
J	>0.001	0.013	>0.001	0.423	>0.001	0.009	0.333	Two-way RM ANOVA, Holm-Sidak post-hoc test
	time	genotype-diet		0 (WT vs KO HFD)	20 (WT vs KO HFD)	40 (WT vs KO HFD)	60 (WT vs KO HFD)	
K	>0.001	0.041	0.023	0.099	0.008	0.021	0.056	Two-way RM ANOVA, Holm-Sidak post-hoc test
	nutrition	genotype		NFD	HFD	genotype	genotype	
				genotype	genotype	NFD vs HFD	NFD vs HFD	
L	>0.001	0.121	0.040	0.222	0.087	0.021	>0.001	Two-way ANOVA, Holm-Sidak post-hoc test
M	>0.001	0.044	0.012	0.121	0.032	0.008	>0.001	Two-way ANOVA, Holm-Sidak post-hoc test
N	>0.001	0.031	0.009	0.257	0.006	>0.001	>0.001	Two-way ANOVA, Holm-Sidak post-hoc test

Figure EV2								
	nutrition	genotype		pre-fast vs fast	pre-fast vs. refed	fast vs refed		
A	>0.001	0.350	0.011	>0.001	0.033	0.222		Two-way RM ANOVA, Holm-Sidak post-hoc test
B	>0.001	0.189	0.008	>0.001	0.027	0.005		Two-way RM ANOVA, Holm-Sidak post-hoc test
C	>0.001	0.675	0.023	0.007	0.009	0.229		Two-way RM ANOVA, Holm-Sidak post-hoc test
D				Gadd45a	Gadd45b	Gadd45g		
				0.448	>0.001	0.664		t-test
	nutrition	genotype		fed	fasted	WT	KO	
				WT vs KO	WT vs KO	fed vs fasted	fed vs fasted	
E	>0.001	0.385	0.012	0.444	0.398	>0.001	>0.001	Two-way ANOVA, Holm-Sidak post-hoc test
H	>0.001	0.251	0.009	0.210	0.206	>0.001	>0.001	Two-way ANOVA, Holm-Sidak post-hoc test
I	0.077	0.222	0.438	NP	NP	NP	NP	Two-way ANOVA, Holm-Sidak post-hoc test
F				Liver	Fat	GCM		
				0.329	0.287	0.401		t-test
G				NEFA	Cholesterol	Glycogen		
				0.201	0.363	0.191		t-test
J			C0	C2	SCAC	MCAC	LCAC	
			0.298	0.329	0.189	0.210	0.427	t-test

Figure S2								
				Gadd45g	Gadd45b			
A				>0.001	0.456			t-test
	nutrition	genotype		fed	fasted	genotype	genotype	
				genotype	genotype	fed vs fasted	fed vs fasted	
B	>0.001	0.284	0.007	0.429	0.321	>0.001	>0.001	Two-way ANOVA, Holm-Sidak post-hoc test
				Liver	pgWAT	BAT	GCM	
C				0.222	0.198	0.267	0.335	t-test
				BG				
D				0.333				t-test
				NEFA	TG	Glycerol	Cholesterol	
E				0.222	0.397	0.442	0.597	t-test
				NEFA	TG	Cholesterol		
F				0.189	0.398	0.523		t-test

Figure S3								
	nutrition	genotype		NFD	HFD	genotype	genotype	
				genotype	genotype	NFD vs HFD	NFD vs HFD	
A	0.345	0.231	0.667	NP	NP	NP	NP	Two-way ANOVA, Holm-Sidak post-hoc test
B	0.287	>0.001	>0.001	>0.001	>0.001	0.324	0.920	Two-way ANOVA, Holm-Sidak post-hoc test
C	0.138	0.409	0.567	NP	NP	NP	NP	Two-way ANOVA, Holm-Sidak post-hoc test
D	>0.001	0.125	>0.001	0.033	0.230	>0.001	>0.001	Two-way ANOVA, Holm-Sidak post-hoc test
E	>0.001	0.238	>0.001	0.099	0.239	>0.001	>0.001	Two-way ANOVA, Holm-Sidak post-hoc test

Figure 4								
	nutrition	genotype		fed/BSA	fasted/BSA-NEFA	WT	KO	
				WT vs KO	WT vs KO	fed vs fasted/BSA vs NEFA	fed vs fasted/BSA vs NEFA	
A	>0.001	0.087	>0.001	0.234	>0.001	0.022	>0.001	Two-way ANOVA, Holm-Sidak post-hoc test
B	>0.001	0.543	>0.001	0.309	0.290	>0.001	>0.001	Two-way ANOVA, Holm-Sidak post-hoc test
C	0.043	0.285	>0.001	0.444	>0.001	0.127	>0.001	Two-way ANOVA, Holm-Sidak post-hoc test
D	>0.001	0.105	>0.001	0.287	>0.001	>0.001	>0.001	Two-way ANOVA, Holm-Sidak post-hoc test
	nutrition	fraction		Hepas	NP	fed	fed	
				fasted vs fed	fasted vs fed	Hepas vs NP	Hepas vs NP	
E	>0.001	0.106	0.025	0.030	0.179	0.088	0.197	Two-way ANOVA, Holm-Sidak post-hoc test
	nutrition	miR		fed	fasted	NC miR	G45b miR	
				NC miR vs G45b miR	NC miR vs G45b miR	fed vs fasted	fed vs fasted	
F	>0.001	0.174	>0.001	0.418	0.026	>0.001	0.005	Two-way ANOVA, Holm-Sidak post-hoc test
G	>0.001	0.201	>0.001	0.317	0.011	0.007	>0.001	Two-way ANOVA, Holm-Sidak post-hoc test
	AD	genotype		NC	G45b OE	WT	KO	
				WT vs KO	WT vs KO	NC vs G45b	NC vs G45b	
H	0.031	0.055	0.012	0.068	0.266	0.087	0.008	Two-way ANOVA, Holm-Sidak post-hoc test
	nutrition	miR		NFD	HFD	NC miR	G45b miR	
				NC miR vs G45b miR	NC miR vs G45b miR	NFD vs HFD	NFD vs HFD	
I	>0.001	0.197	0.023	0.314	0.451	0.005	>0.001	Two-way ANOVA, Holm-Sidak post-hoc test
J	>0.001	0.182	>0.001	0.202	0.028	>0.001	>0.001	Two-way ANOVA, Holm-Sidak post-hoc test
K	>0.001	0.213	>0.001	0.438	0.024	>0.001	>0.001	Two-way ANOVA, Holm-Sidak post-hoc test

Figure EV3								
	nutrition	fraction		Hepas	NP	fed	fed	
				fasted vs fed	fasted vs fed	Hepas vs NP	Hepas vs NP	
A	0.444	0.014	0.105	NP	NP	NP	NP	Two-way ANOVA, Holm-Sidak post-hoc test
B	0.600	>0.001	0.104	NP	NP	NP	NP	Two-way ANOVA, Holm-Sidak post-hoc test
C	0.537	>0.001	0.283	NP	NP	NP	NP	Two-way ANOVA, Holm-Sidak post-hoc test
D	0.329	>0.001	0.406	NP	NP	NP	NP	Two-way ANOVA, Holm-Sidak post-hoc test
	nutrition	miR		fed	fasted	NC miR	G45b miR	
				NC miR vs G45b miR	NC miR vs G45b miR	fed vs fasted	fed vs fasted	
E	>0.001	>0.001	>0.001	0.003	>0.001	>0.001	**0.037**	Two-way ANOVA, Holm-Sidak post-hoc test
	AD	genotype		NC	G45b OE	WT	KO	
				WT vs KO	WT vs KO	NC vs G45b	NC vs G45b	
F	>0.001	0.105	>0.001	>0.001	0.211	>0.001	>0.001	Two-way ANOVA, Holm-Sidak post-hoc test
G	0.229	0.381	0.449	NP	NP	NP	NP	Two-way ANOVA, Holm-Sidak post-hoc test
H	0.294	0.087	0.009	0.223	>0.001	0.276	>0.001	Two-way ANOVA, Holm-Sidak post-hoc test
	nutrition	miR		NFD	HFD	NC miR	G45b miR	
				NC miR vs G45b miR	NC miR vs G45b miR	NFD vs HFD	NFD vs HFD	
I	>0.001	0.041	>0.001	0.319	0.033	>0.001	>0.001	Two-way ANOVA, Holm-Sidak post-hoc test
J	>0.001	0.143	>0.001	0.252	0.128	>0.001	>0.001	Two-way ANOVA, Holm-Sidak post-hoc test
K	>0.001	0.261	>0.001	0.278	0.124	>0.001	>0.001	Two-way ANOVA, Holm-Sidak post-hoc test

Figure 5								
	AD	genotype		NC	G45b OE	WT	db/db	
				WT vs db/db	WT vs db/db	NC vs G45b	NC vs G45b	
A	0.030	>0.001	>0.001	>0.001	0.038	0.081	>0.001	Two-way ANOVA, Holm-Sidak post-hoc test
B	0.201	>0.001	>0.001	>0.001	0.061	0.286	0.028	Two-way ANOVA, Holm-Sidak post-hoc test
C	0.044	>0.001	>0.001	>0.001	0.092	0.099	>0.001	Two-way ANOVA, Holm-Sidak post-hoc test
		NGT vs T2D						
D		>0.001						Mann-Whitney Rank Sum Test

Figure EV4								
	AD	genotype		NC	G45b OE	WT	db/db	
				WT vs db/db	WT vs db/db	NC vs G45b	NC vs G45b	
A	0.005	0.150	0.065	NP	NP	NP	NP	Two-way ANOVA, Holm-Sidak post-hoc test
B	0.122	>0.001	>0.001	>0.001	>0.001	0.124	0.048	Two-way ANOVA, Holm-Sidak post-hoc test
C	0.228	0.356	0.437	NP	NP	NP	NP	Two-way ANOVA, Holm-Sidak post-hoc test
D	0.264	>0.001	0.008	>0.001	>0.001	0.187	0.235	Two-way ANOVA, Holm-Sidak post-hoc test

Figure 6										
	AD	genotype		NC	G45b OE	WT	db/db			
				WT vs KO	WT vs KO	NC vs G45b	NC vs G45b			
A	>0.001	0.085	>0.001	0.019	0.205	>0.001	>0.001	Two-way ANOVA, Holm-Sidak post-hoc test		
B										
Slc27a2	0.305	0.351	0.555	NP	NP	NP	NP	Two-way ANOVA, Holm-Sidak post-hoc test		
Slc27a5	0.225	0.394	0.312	NP	NP	NP	NP	Two-way ANOVA, Holm-Sidak post-hoc test		
Cd36	0.033	0.210	0.267	NP	NP	NP	NP	Two-way ANOVA, Holm-Sidak post-hoc test		
Fabp1	0.333	0.259	0.310	NP	NP	NP	NP	Two-way ANOVA, Holm-Sidak post-hoc test		
Dbi	0.158	0.159	0.426	NP	NP	NP	NP	Two-way ANOVA, Holm-Sidak post-hoc test		
Acly	0.204	0.194	0.257	NP	NP	NP	NP	Two-way ANOVA, Holm-Sidak post-hoc test		
Dgat1	0.242	0.525	0.316	NP	NP	NP	NP	Two-way ANOVA, Holm-Sidak post-hoc test		
Atgl	0.328	0.310	0.428	NP	NP	NP	NP	Two-way ANOVA, Holm-Sidak post-hoc test		
Hsl	0.555	0.364	0.292	NP	NP	NP	NP	Two-way ANOVA, Holm-Sidak post-hoc test		
I	0.222	0.109	>0.001	>0.001	0.289	0.346	>0.001	Two-way ANOVA, Holm-Sidak post-hoc test		
				MS	CYTO	WT	KO			
	fraction	genotype		WT vs KO	WT vs KO	MS vs CYTO	MS vs CYTO			
D	>0.001	>0.001	>0.001	>0.001	>0.001	>0.001	0.206	Two-way ANOVA, Holm-Sidak post-hoc test		
E				WT-NC vs KO-NC	WT-NC vs WT-OE	KO-NC vs KO-OE	WT-OE vs KO-OE			
WTL				0.444	0.413	0.358	0.333	One-way ANOVA, Holm-Sidak post-hoc test		
MS				>0.001	0.210	>0.001	0.458	One-way ANOVA, Holm-Sidak post-hoc test		
CYTO				>0.001	0.341	>0.001	0.297	One-way ANOVA, Holm-Sidak post-hoc test		
				MS	CYTO	NC	G45b			
	fraction	genotype		NC vs G45b	NC vs G45b	MS vs CYTO	MS vs CYTO			
F	0.205	0.168	>0.001	>0.001	>0.001	0.305	>0.001	Two-way ANOVA, Holm-Sidak post-hoc test		
	genotype									
H	>0.001								t-test	
	AD	genotype		NC	G45b OE	WT	db/db			
				WT vs db/db	WT vs db/db	NC vs G45b	NC vs G45b			
J	0.093	>0.001	0.015	>0.001	0.008	0.241	>0.001	Two-way ANOVA, Holm-Sidak post-hoc test		

Figure EV5									
	AD								
A	0.524								t-test
B	0.412								t-test
D	genoytpe								
LC3B	0.242								t-test
p-S6K1	0.358								t-test
p-ERK	0.153								t-test
p-p38	0.204								t-test
p-PERK	0.123								t-test
p-eIF2a	0.423								t-test
FATP2	0.334								t-test
CD36	0.267								t-test
FABP1	0.346								t-test
