# Supplementary material for: Fasting‐induced liver GADD45β restrains hepatic fatty acid uptake and improves metabolic health
Source: EMBO Mol Med. 2016 May 3;8(6):654–69. doi: 10.15252/emmm.201505801 (PMC4888855; doi:10.15252/emmm.201505801)
Supplement: Supplementary file 2 — Expanded View Figures PDF [file EMMM-8-654-s002.pdf]

Expanded View Figures

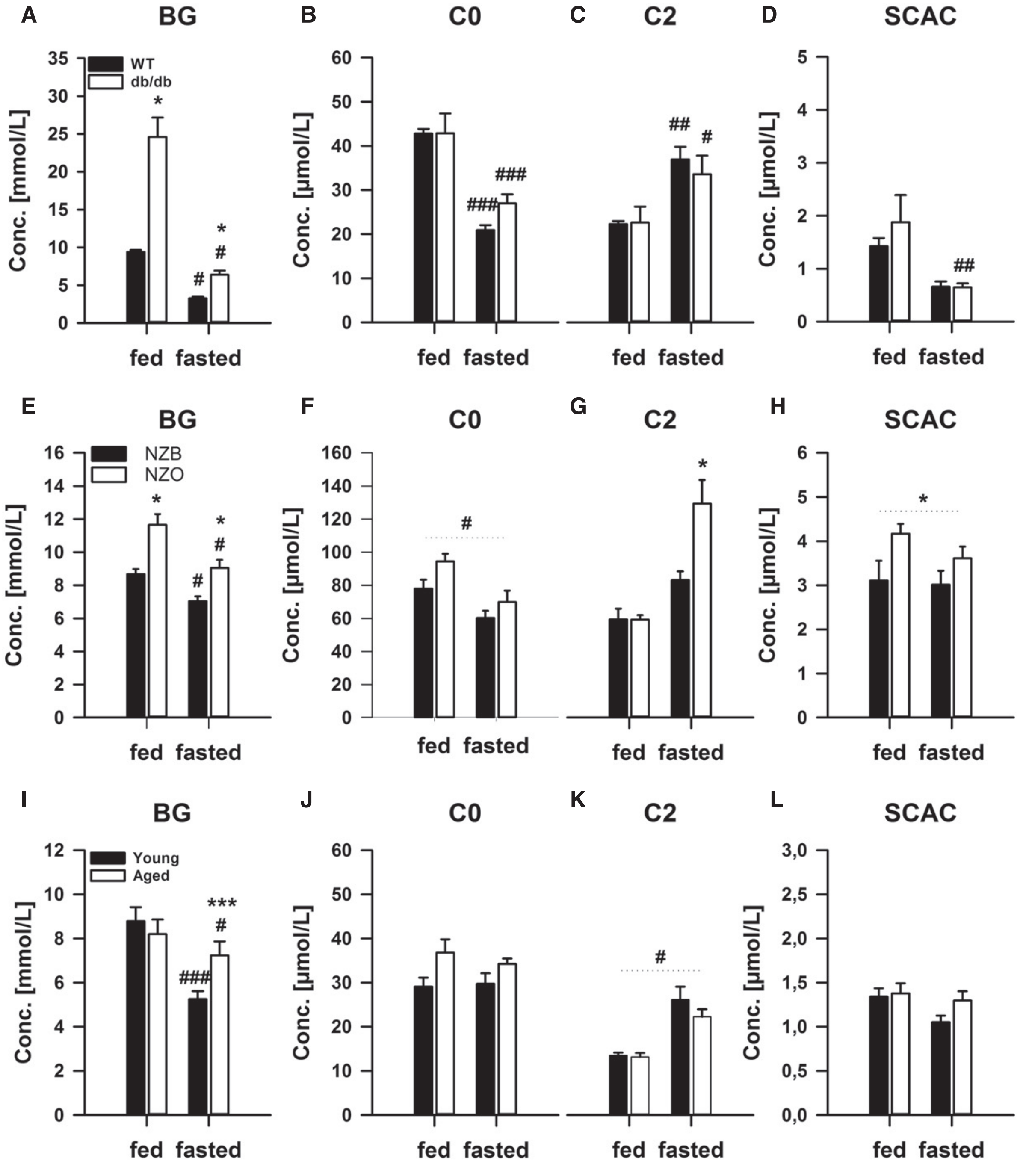

Figure EV1. The dysregulated lipid metabolic phenotype of mouse models of metabolic dysfunction is most pronounced in the fasted state.

A–L Male 12-weeks-old wild-type (WT; C57Bl/6J) or obese/diabetic monogenic (*db/db*; BKS.Cg-$m^{+/+}$ Lepr DB/J; $n = 4$/group, A–D), New Zealand Black (NZB) and polygenic obese/pre-diabetic New Zealand Obese (NZO; $n = 4$/group; E–H), as well as young (i.e. 3 months) and aged (i.e.22 months; $n = 5$/group; I–L), mice were fed *ad libitum* (fed) or fasted for 24 h (fasted). Blood glucose (BG, A, E, I) and serum acylcarnitine species including free carnitine (C0; B, F, J), acylcarnitine (C2; C, G, K) and short-chain acylcarnitines (SCAC; D, H, L) were measured. Data are mean ± SEM. $n = 4$/group. Effect of genotype, *$P < 0.05$, **$P < 0.01$, ***$P < 0.001$. Effect of nutritional state: $^{\#}P < 0.05$, $^{\#\#}P < 0.01$, $^{\#\#\#}P < 0.001$. The statistical test used and respective *P*-value outputs can be found in Appendix Table S1.

© 2016 The Authors

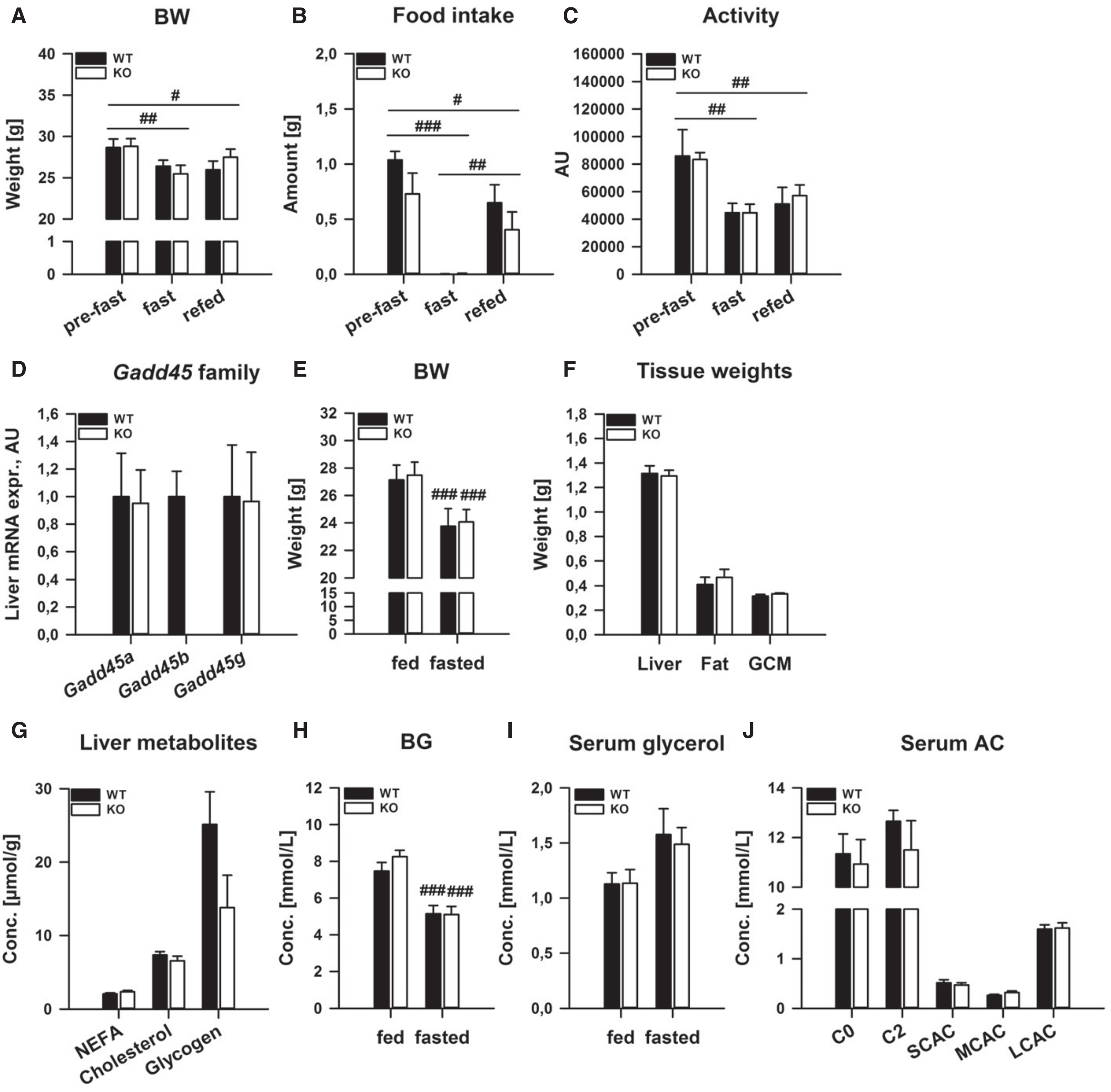

Figure EV2. Systemic GADD45β deletion affects metabolic regulation under conditions of heightened lipid metabolism.

A–C Male GADD45β$^{+/+}$ (WT) or GADD45β$^{-/-}$ (KO) were fed *ad libitum* (fed) or fasted for 24 h (fasted), and subsequently refed for 24 h (n = 4–6/group). Body weight (A), food intake (B) and physical activity (C) were measured.

D–J In a distinct cohort, male GADD45β$^{+/+}$ (WT) or GADD45β$^{-/-}$ (KO) mice were fasted for 24 h (fasted) (n = 5–8/group) with blood samples taken before and during fasting. Liver mRNA expression of *Gadd45* family members (D). Body weight (E) before and after fasting and fasted tissue weights (F). Selected liver metabolites (G) as well as blood glucose (H) and serum glycerol (I) and acylcarnitines (J).

Data information; Data are mean ± SEM. Effect of genotype, *$P < 0.05$, **$P < 0.01$, ***$P < 0.001$. Effect of nutritional state: $^{\#}P < 0.05$, $^{\#\#}P < 0.01$, $^{\#\#\#}P < 0.001$. The statistical test used and respective P-value outputs can be found in Appendix Table S1.

© 2016 The Authors

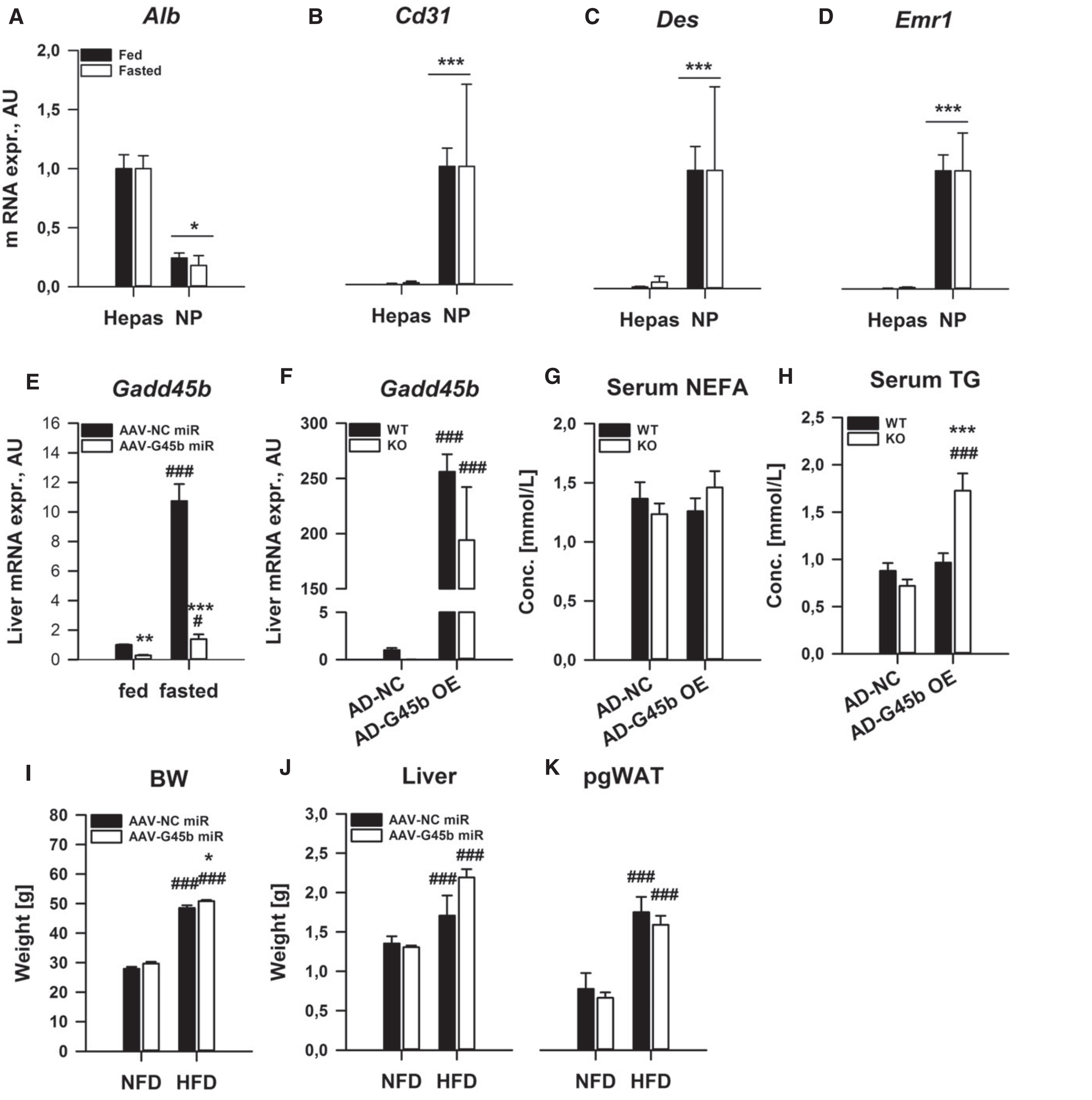

Figure EV3. Liver-restricted GADD45β manipulation affects systemic metabolic homoeostasis.

A–D Albumin (A), Cluster of differentiation 31 (B), desmin (C) and EGF-like module-containing mucin-like hormone receptor-like 1 (D) mRNA expression were measured from fractionated parenchymal hepatocytes (Hepas) as well as non-parenchymal cells (NP) from C57Bl/6J mice fed *ad libitum* (fed) or fasted for 24 h (fasted) (n = 3/group).

E Male C57Bl/6J mice with (AAV-G45b miR) or without (AAV-NC miR) liver/hepatocyte-restricted GADD45β silencing were fed or fasted for 24 h (n = 6/group). Liver Gadd45b mRNA expression was measured.

F–H Male GADD45β$^{+/+}$ (WT) or GADD45β$^{-/-}$ (KO) mice fasted for 24 h (fasted) with (AD-G45b OE) or without (AD-NC) liver-restricted Gadd45b over-expression (n = 7–8/group). Liver mRNA expression of *Gadd45b* (F). Serum non-esterified fatty acids (G) and triglycerides (H) were measured.

I–K Male C57Bl/6J mice with (AAV-G45b miR) or without (AAV-NC miR) liver/hepatocyte-restricted GADD45β silencing were chronically fed a normal-fat diet (NFD) or high -fat diet (HFD) and were sacrificed in the *ad libitum* fed state (n = 6–8/group). Body (I), liver (J) and perigonadal white adipose tissue (K) masses.

Data information: Data are mean ± SEM. Effect of genotype, $^*P < 0.05$, $^{**}P < 0.01$, $^{***}P < 0.001$. Effect of nutritional state: $^{\#}P < 0.05$, $^{\#\#}P < 0.01$, $^{\#\#\#}P < 0.001$. The statistical test used and respective P-value outputs can be found in Appendix Table S1.

© 2016 The Authors

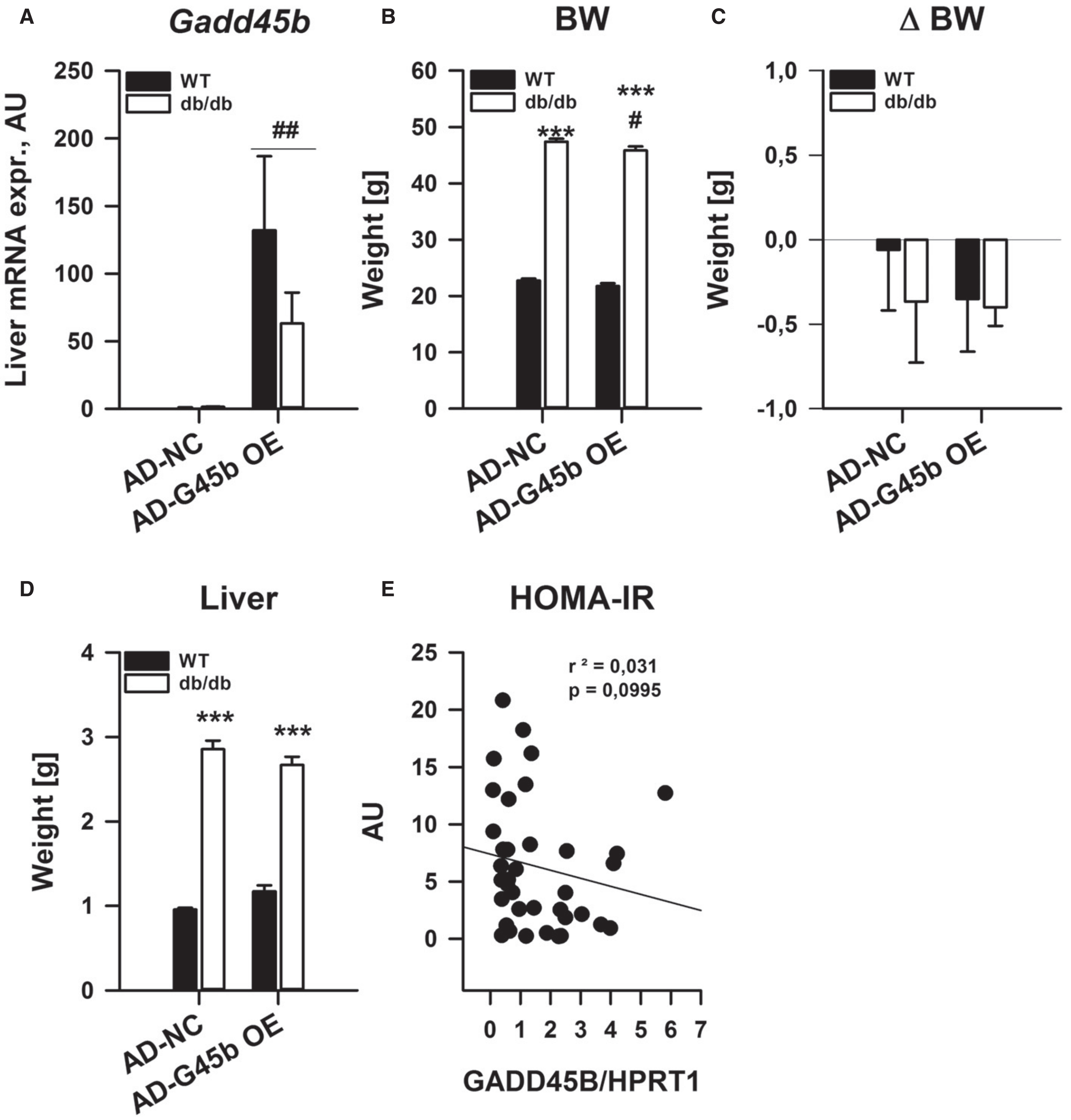

Figure EV4. Liver GADD45β expression modulates metabolic control in type 2 diabetes.

A–D Male 12-weeks-old wild-type (WT; C57Bl/6J) or obese/diabetic (*db/db*; BKS.Cg-$m^{+/+}$ Lepr DB/J) mice with (AD-G45b OE) or without (AD-NC) liver-restricted GADD45β over-expression were fasted for 24 h (n = 4–6/group). Liver mRNA expression of *Gadd45b* (A). Body mass (B), the change in body mass during the experiment (C) and liver mass (D). Data are mean ± SEM. Effect of genotype, *$P < 0.05$, **$P < 0.01$, ***$P < 0.001$. Effect of viral manipulation state: $^{\#}P < 0.05$, $^{\#\#}P < 0.01$, $^{\#\#\#}P < 0.001$.

E Fasting HOMA-IR in correlation with liver *GADD45B* mRNA expression in men as in Fig 5 (n = 37). Inserts show r^2 values and P-values from Spearman's correlation test.

Data information: The statistical test used and respective P-value outputs can be found in Appendix Table S1.

 © 2016 The Authors

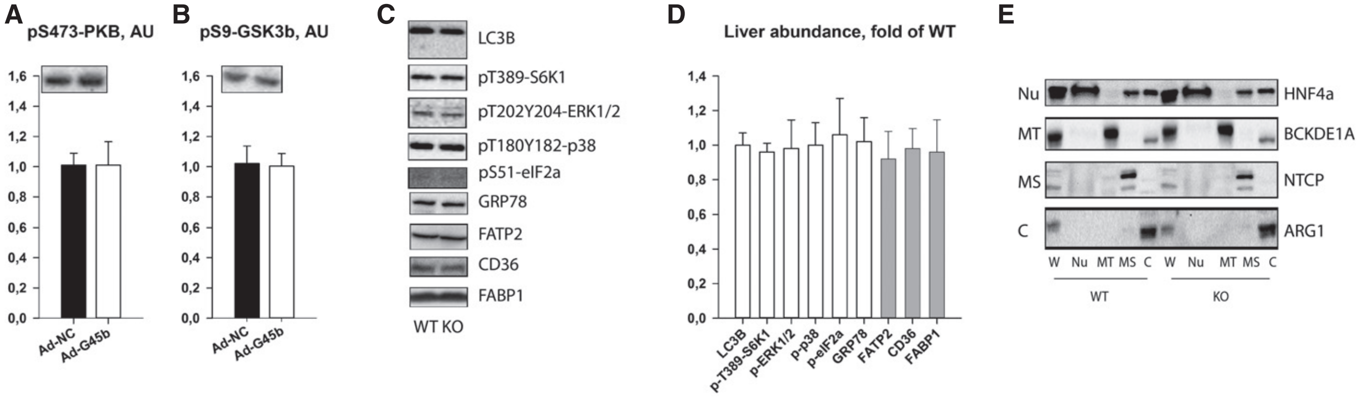

Figure EV5. Liver GADD45β controls liver fatty acid handling by cytosolic FABP1 retention.

A, B Male 12-weeks-old wild-type (WT; C57Bl/6J) or obese/diabetic (*db/db*; BKS.Cg-$m^{+/+}$ Lepr DB/J) mice with (Ad-G45b OE) or without (Ad-NC) prior liver-restricted GADD45β over-expression were fasted and insulin was injected with livers harvested shortly thereafter and subsequently liver proteins were subjected to immunoblotting for insulin signalling proteins including phosphoprotein kinase B (PKB/Akt; A) and glycogen synthase kinase beta (GSK3b; B). Inserts show representative blots (n = 6/group).

C, D Representative immunoblots (C) and relative abundance quantifications (D; n = 6) of proteins and phosphoproteins including light chain 3 isoform B (LC3B), S6 kinase 1 (S6K1), p42/44 mitogen activated protein kinase (ERK1/2), eukaryotic initiation factor 2 alpha (eIF2a), glucose regulated protein 78 (GRP78/HSPA5), fatty acid transport protein 2 (FATP2/SLC27A2), cluster determinant 36 (CD36/FAT) and fatty acid binding protein 1 (FABP1) from fasted GADD45$\beta^{+/+}$ (WT) or GADD45$\beta^{-/-}$ (KO) mice.

E Representative immunoblots of HNF4a (nuclear marker), BCKDE1A (mitochondrial marker), NTCP (microsomal marker) and ARG1 (cytosolic marker) from liver whole tissue lysate (W) as well as fractionated organelles/intraceuular structures including nuclei (N), mitochondria (MT), microsomes (MS) and cytoplasm (C), from GADD45$\beta^{+/+}$ (WT) and GADD45$\beta^{-/-}$ (KO) mice.

Data information: Data are mean ± SEM. The statistical test used and respective *P*-value outputs can be found in Appendix Table S1.

© 2016 The Authors
